# Regime shift dynamics, tipping points and the success of fisheries management

**DOI:** 10.1038/s41598-022-27104-y

**Published:** 2023-01-06

**Authors:** Alexandra M. Blöcker, Helene M. Gutte, Reuven L. Bender, Saskia A. Otto, Camilla Sguotti, Christian Möllmann

**Affiliations:** 1 Center for Earth System Research and Sustainability (CEN), Institute of Marine Ecosystem and Fisheries Science (IMF), University of Hamburg, 22767 Hamburg, Germany; 2 Department of Biology, University of Padova, Via Bassi, 35100 Padova, Italy

**Keywords:** Climate-change ecology, Marine biology

## Abstract

Recovery of depleted fish stocks is an important goal for fisheries management and crucial to sustain important ecosystem functions as well as global food security. Successful recovery requires adjusting fishing mortality to stock productivity but can be prevented or inhibited by additional anthropogenic impacts such as climate change. Despite management measures to recover fish stocks being in place in legislations such as the European Union´s Common Fisheries Policy (CFP), recovery can be hindered by the occurrence of regime shift dynamics. Such non-linear discontinuous dynamics imply tipping points and bear the characteristics of abrupt change, hysteresis and non-stationary functional relationships. We here used the recent reform of the CFP as a natural experiment to investigate the existence of regime shift dynamics and its potential effects on the recovery potential on six strongly fished or even depleted commercial fish stocks in the North Sea. Using a set of statistical approaches we show that regime shift dynamics exist in all six fish stocks as a response to changes in fishing pressure and temperature. Our results furthermore demonstrate the context-dependence of such dynamics and hence the ability of management measures to rebuild depleted fish stocks, leading to either failed recovery or positive tipping.

## Introduction

Recovery of depleted fish stocks is an important goal for fisheries management and crucial to sustain important ecosystem functions as well as global food security^1^. Successful recovery requires adjusting fishing mortality to stock productivity but can be prevented or inhibited by additional anthropogenic impacts such as climate change, e.g. through temperature increase^2^. In the European Union (EU), fisheries management aims at fish stock recovery through regulations embedded in the so called common fisheries policy (CFP), first implemented in 1983, to enhance the management of fisheries and fish stocks. The CFP underwent several successive reforms (the latest in 2014) and aims for a fishery that is environmentally, economically and socially sustainable^1^. One of the main features of the CFP is the introduction of the Maximum Sustainable Yield (MSY) concept, where MSY is defined as “the highest theoretical equilibrium yield that can be continuously taken from a stock under existing average environmental conditions without significantly affecting the reproduction process”^1^. In EU fisheries management MSY is implemented through a target fishing mortality F_MSY_ (fishing mortality level aiming at Maximum Sustainable Yield)^3^ and MSY B_trigger_, a limit reference value below which F_MSY_ is adjusted^4^. Annual stock assessments advising EU fisheries management are provided by the International Council for the Exploration of the Sea (ICES) that, among others, provides reconstructed time-series of fishing mortality (F), spawning stock biomass (SSB, the parent biomass) and recruitment of the stock (R, size at the incoming new year-class)^3^.

Within the EU, the North Sea is among the most heavily impacted areas of the world´s oceans suffering from diverse anthropogenic activities^5^. It was a hotspot of overfishing with several fish stocks suffering from unsustainable fishing pressure, and the collapse of the Atlantic cod being a prominent example^2,5^. Fishing pressure was reduced strongly to prevent further depletion of fish stocks, but as with cod, recovery was not successful for all species^2^. In addition, the North Sea is currently considered a hot spot of climate change experiencing rapid warming and acidification, affecting the distribution of the North Sea fish community^5^. Cold water species experience a shift northwards, whereas subtropical species like sardine and anchovy appear more likely^6–8^. Hence, as many commercially important fish stocks in the North Sea experienced overexploitation and climate change effects are increasing continuously, the effective implementation of the EU fisheries management to achieve sustainable reference levels is crucial for stock recovery^2^.

Despite management measures to recover fish stocks being in place, recovery can be hindered by the occurrence of non-linear discontinuous dynamics of ecological systems^2,9,10^. Such dynamics imply tipping points, where, e.g., a fish stock’s biomass crosses a critical threshold at which two dynamic regimes can be separated, the so-called alternative states; a high biomass state above MSY and an unsustainable low biomass state. Hence, a tipping point is defined as an abrupt change in the dynamics in response to changes in internal or external pressures^11^. The concept of tipping points usually implies discontinuous regime shifts of systems and includes three characteristics: (1) abrupt change, (2) hysteresis, and (3) non-stationary functional relationships^11–13^. Abrupt change is the first-order indicator of regime shifts. According to regime shift theory such changes occur when an external pressure, e.g. fishing mortality, exceeds a threshold or through the interaction of drivers like fishing pressure and temperature, where changes in one pressure can modify the interaction between the driver and the state variable and induce the shift towards an alternative state^11–13^. Abrupt changes do not necessarily imply discontinuous dynamics and additional analyses to detect underlying drivers are required^14^. If an external pressure is reduced and the return path from the new alternative to the original state is different from the path that led to the new state, hysteresis is present. If hysteresis or even irreversibility occurs, often two drivers interact in causing non-stationary functional relationships^11–13^. A non-stationary relationship implies e.g. shifts in the so-called stock-recruitment relationship (SRR). SRR is the most important relationship in fish stock dynamics and relates the SSB to R; both being essential to determine allowable catches^15^. Non-stationarity in the SRR changes the relationship between SSB and R, where, in the worst case, R is no longer determined by SSB^16^. Hence, knowing the stock’s SRR is crucial for a sustainable fisheries management^15^.

We here used the recent reform of the CFP as a natural experiment to investigate the existence of non-linear discontinuous dynamics and its potential effects on the recovery potential of strongly fished or even depleted commercial fish stocks in the North Sea. We focused on a selection of important North Sea fish stocks since this region has experienced several regime shifts, and is a focus area of marine regime shift science^17^. Therefore we assessed the effectiveness of reducing fishing mortality to sustainable reference levels and potential recovery patterns of European plaice (*Pleuronectes platessa*), European hake (*Merluccius merluccius*), Atlantic herring (*Clupea harengus*), haddock (*Melanogrammus aeglefinus*), saithe (*Pollachius virens*), and Atlantic cod (*Gadus morhua*). These species are commercially relevant and cover a range of life-history strategies and taxonomic groups, i.e. pelagic and demersal as well as round and flat fishes. Here, we used fish stock (SSB, R) and fishing mortality (F) data from the International Council for the Exploration of the Sea (ICES) in the ICES Stock Assessment Database (data extracted in 2021)^18^.

Our study revealed regime shift characteristics to exist in all six North Sea fish stocks investigated, suggesting the prevalence of discontinuous dynamics in exploited living marine resources. Our results furthermore demonstrate the context-dependence of such dynamics and hence the ability of management measures to rebuild depleted fish stocks, leading to either failed recovery or positive tipping.

## Results

### Temporal dynamics and abrupt changes

**Figure 1:**
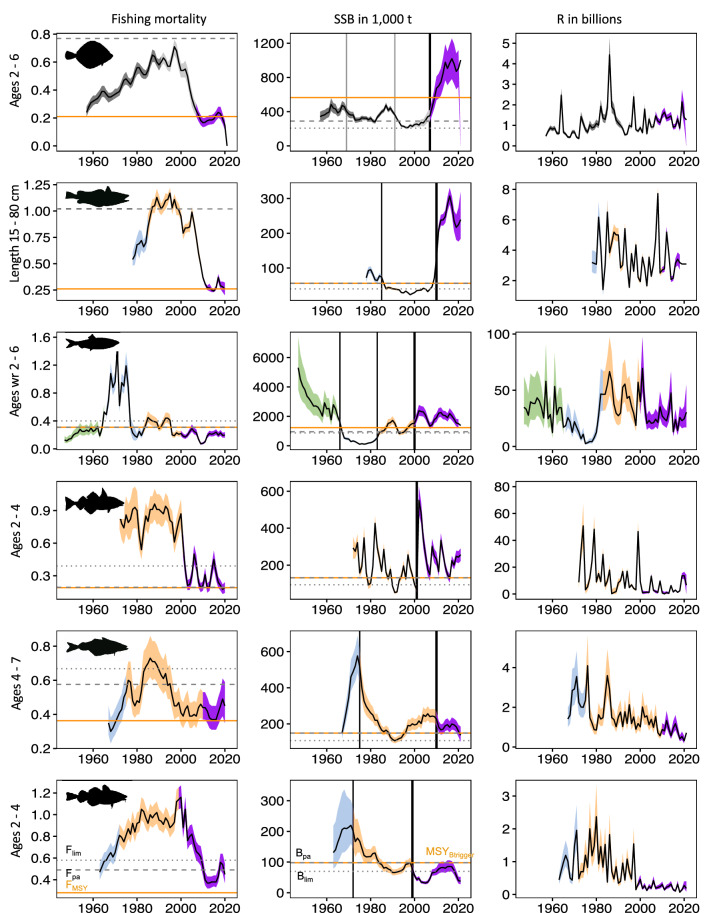
Abrupt changes in North Sea fish stocks. Time series of fishing mortality (F), spawning stock biomass (SSB) and recruitment (R). Black vertical lines in SSB indicate abrupt changes detected by both statistical change point analyses. Grey ranges and vertical lines in SSB for plaice indicate abrupt changes detected by the *BinSeg* algorithm only. Coloured ranges show identified SSB periods (see also Table 1); horizontal lines indicate management reference points; left column—F level aiming at Maximum Sustainable Yield (F_MSY_), precautionary level of F (F_pa_), limit reference point of F (F_lim_); middle column—Level of SSB triggering specific action in management (MSY B_trigger_), precautionary level of SSB (B_pa_), limit reference point of SSB (B_lim_)^3^.

We first analysed time-series of fishing mortality (F), spawning stock biomass (SSB) and recruitment (R) to compare stock development and status among the target fish stocks of our study and to identify potential abrupt changes. For the six North Sea fish stocks, F was periodically excessively high (even > 1.0), i.e. far above the present F_MSY_ target (Fig. 1—left column). This high fishing mortality lasted until the beginning of the 2000s (and partly longer) for plaice, hake, haddock and cod. During the early 2000s EU fisheries management succeeded to reduce F for all species, except for cod, to levels at or close to F_MSY_. Plaice and hake responded immediately with strong increases to unprecedented levels in SSB, far above the limit reference level (MSY B_trigger_; the threshold SSB triggering a reduction in F_MSY_^3^) (Fig. 1—middle column). However, low responses in SSB to decreasing fishing mortalities are observed for the remaining species, in haddock only noticeable with a peak year. Importantly, cod remains exclusively below MSY B_trigger_.

Statistical change point analyses (see Methods) revealed several abrupt changes, the first flag indicating regime shift dynamics in SSB (Table 1; Fig. 1—middle column, all change points in Supplementary Table S1). For plaice and herring, three abrupt changes separating four periods were found, whereas only one abrupt change was found for haddock. For plaice, the former three abrupt changes were only detected by the *BinSeg* algorithm embedded in the changepoint package. The strong increase in SSB in the last period might mask these smaller changes for the Bayesian approach calculating the posterior probabilities of changes, embedded in the bcp package. The identified periods for plaice and herring overlap strongly (decades: 1950s–60s, 1970s–80s, 1980s–2000s, 2000s–2010s) and change points only differ by 8 years in maximum. Also, the periods of saithe and cod were almost in synchrony, except for the last period where the change occurred 11 years earlier for cod (decades: 1960s–70s, 1970s–2000s, 2000s–2010s). These abrupt changes preceded collapses for saithe and cod, while plaice and hake showed a positive stock development following the last abrupt change.

**Table 1:** Abrupt changes in North Sea fish stocks.

Species	Period 1	Period 2	Period 3	Period 4
Plaice	1957–1969	1970–1991	1992–2007	2008–2021
Hake	1978–1985	1986–2010	2011–2021	
Herring	1947–1966	1967–1983	1984–2000	2001–2021
Haddock	1965–2001	2002–2021		
Saithe	1967–1975	1976–2010	2011–2021	
Cod	1963–1972	1973–1999	2000–2021	

Periods of quasi-stability in spawning stock biomass (SSB) identified by statistical change point analyses.

Apart from SSB, R is an important determinant of a population’s success and a major determinant of the dynamics of commercially important fish. The number of juveniles potentially joining the parent’s biomass eventually determines the size of SSB, whereas the size of SSB affects the size of recruitment (SRR)^19,20^. Whether the influence of SSB on R is greater or vice versa, depends on the stock status; depleted stocks like North Sea cod experience a higher influence of SSB on R, with R following SSB patterns^19^. Hence, we inspected the recruitment dynamics relating them to changes in SSB (Fig. 1—right column). R was relatively stable over time in plaice and increased only slightly towards the end of the time series, which is likely a response to increased SSB. For herring, recruitment development appears to follow SSB patterns, with high R at high SSB levels and vice versa. R in hake remained largely unchanged regardless of SSB development. For the remaining stocks (saithe and cod), recruitment decreased strongly simultaneously with SSB decrease. Small occasional increases in SSB in these stocks did not lead to any improvement in R.

### Hysteresis

**Figure 2:**
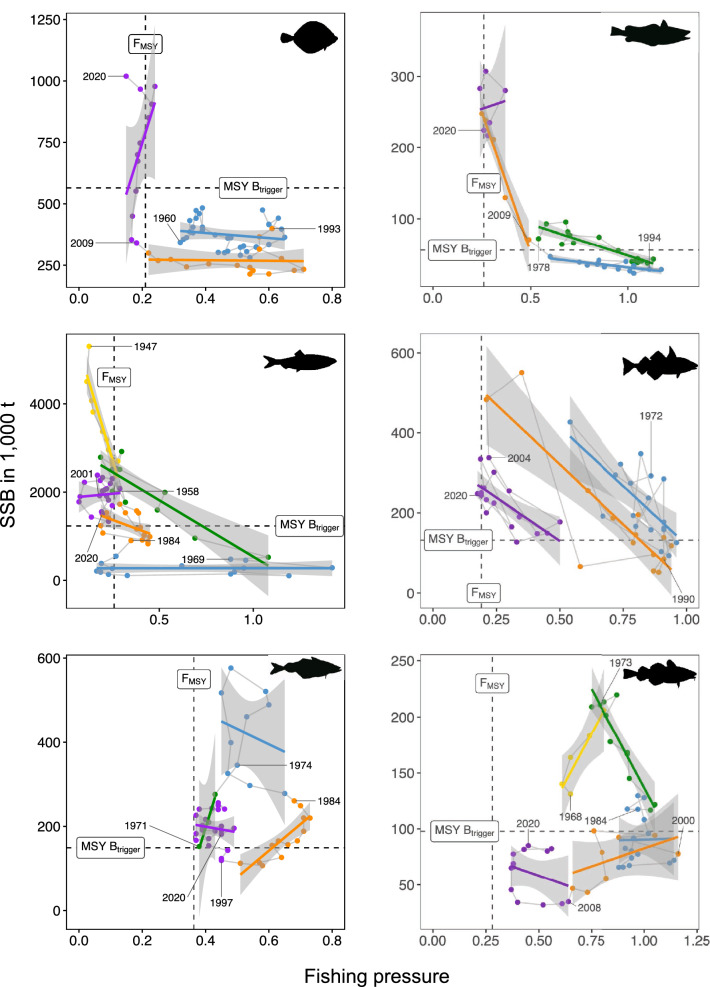
Hysteresis in North Sea fish stocks. Vertical and horizontal lines show sustainable fishing pressure (F_MSY_) and the level of spawning stock biomass (SSB) triggering specific action in management (MSY B_trigger_), respectively. Coloured points show SSB periods identified by break point analyses (see also Table 1). Time lags (F_t−n_) of 3, 4, and 5 years were used for plaice, saithe and cod, respectively.

To determine whether previously identified abrupt changes in SSB and related R patterns do reflect regime shift like dynamics, we studied the existence of hysteresis in the selected North Sea fish stocks, i.e. if the return path in population size (spawning stock biomass—SSB) is different after reduction of the pressure (fishing mortality—F) compared to the preceding path to depletion (the second regime-shift flag)^11–13^. Hence, by relating SSB to F we can identify hysteresis given two different paths of SSB towards and away from depletion. The cross-correlation function revealed that plaice, saithe and cod require time lags (F_t−n_) of 3, 4, and 5 years, respectively. Breakpoints were found in all F-SSB relationships (Fig. 2, all break points in Supplementary Table S1), and closely align with the periods found in the SSB time series (Fig. 1). We observed similar patterns in all species, except for haddock. For plaice, saithe and cod a positive relationship is observed during the first time periods. As F increased in the following periods, the F-SSB relationship became negative, as observed for hake and herring in their first periods. When F was reduced to F_MSY_, a negative relationship at a lower SSB close to MSY B_trigger_ is seen for hake, saithe and cod. Here, herring showed a decoupling from F (blue period). Whereas saithe and cod each experience negative relationships at the end of the time series, plaice and hake show again positive relationships with further decreases in F. These observed changes in the F-SSB relationships for plaice, hake, herring, saithe and cod clearly indicate hysteresis dynamics.

### Non-stationary functional relationships

An important mechanistic criterium for regime shifts dynamics is non-stationarity in important functional relationships governing a dynamic system (the third regime-shift flag)^16^. SSB and R are the two most important interacting attributes of fish stocks^15^. Still, the links between these two are not yet fully understood and can be disrupted by external factors, such as temperature^16^. Therefore, we here studied the stock-recruitment relationships (SRR) to determine the potential existence of non-stationarity. We compared traditional and continuous SRR models (i.e. Ricker and Beverton and Holt models) to a linear model and models incorporating a sudden structural change in the effect of SSB on R (see Methods). In all cases the discontinuous models provided better fits than the traditional and the linear models (comparison of models in Supplementary Table S2) indicating non-stationarity for all stocks.

**Figure 3:**
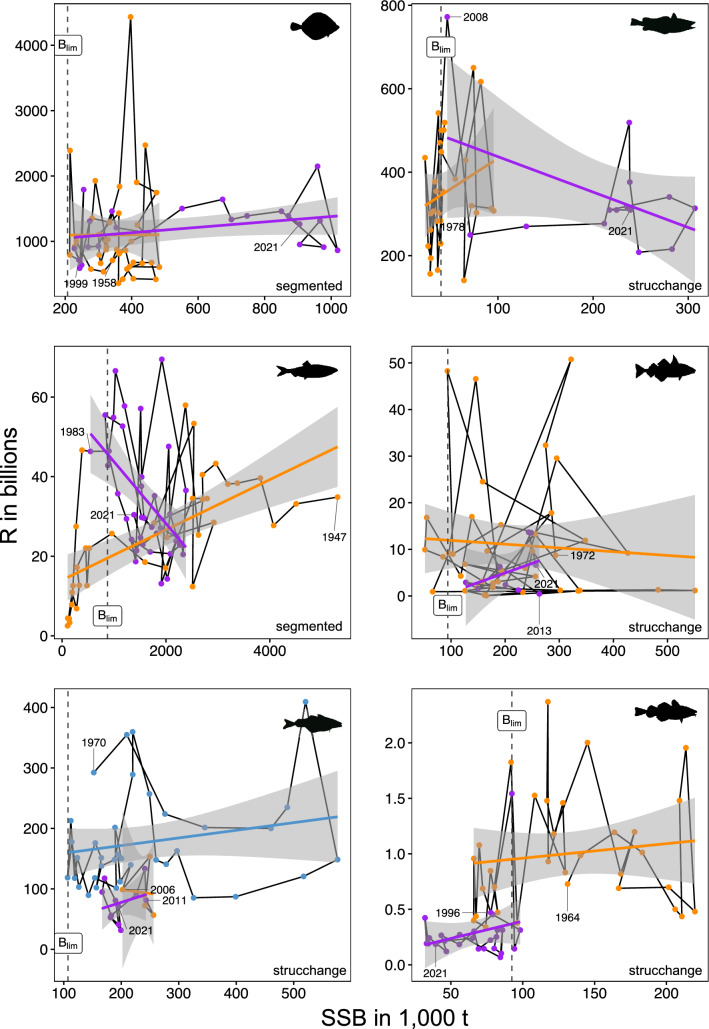
Non-stationary functional relationships in North Sea fish stocks. Break points in non-linear stock-recruitment relationships. Vertical dashed line represents biomass reference level B_lim_. Colours represent linear models per periods identified by breakpoint analyses.

We found for all SRRs an abrupt break point (for saithe two break points), at which the SRR between SSB and R changed (Fig. 3, all break points in Supplementary Table S1) and cover overlapping SSB values. Especially hake, herring and haddock show contrasting relationships, with hake following the typical density-dependence at which the SRR was positive before and negative after break point. Usually, the SRR is dome-shaped and show low R levels at high SSB levels as in the Beverton–Holt and Ricker curves^19^. Especially cod still shows very low SSB values, below B_lim_, and low R values in recent years and despite a slight increase in the past 5 years in SSB, R did not increase. This break point in the late 1990s has also been found in our change point analyses.

Knowing that the North Sea is a climate change hot spot with increasing water temperatures^5^, we explored whether warming has an effect on the SRR. We used Generalized Additive Models (tGAMs; see Methods) that are able to identify a threshold of a pressure like sea surface temperature (SST) at which the relationship between SSB and R is affected. A significant threshold indicates that certain levels of SST can change the SRR shown by differences in the slope^21^.

Our Leave-One-Out Cross-Validation (LOOCV) analysis revealed only one tGAM model with a significant temperature threshold for saithe (Supplementary Table S3, Supplementary Fig. 1), indicating that SST causes a slightly different effect in the SRR before and after the threshold (10.2 °C). SSB has a strong positive effect on R before the threshold, which is less strong after the threshold.

## Discussion

In our study we used the recent reform of the European Union´s Common Fisheries Policy (CFP) as a natural experiment to investigate the existence of regime shift dynamics in North Sea fish stocks, and their implications for achieving management and especially rebuilding targets. We found in all investigated fish species abrupt changes in SSB indicating regime shift dynamics. Abrupt changes were mostly responses to changes in fishing pressure^22–28^ interacting with environmental changes such as resulting from climate change and the positive effects of reduced eutrophication of the North Sea^23,29,30^. In all fish species, except haddock, we additionally identified hysteresis effects, differing in strength. With the exception of cod, all investigated North Sea fish species responded to reduced fishing mortality as a result of implementing the MSY concept in the CFP. Hence sustainable stock sizes were achieved, but the recovery occurred slower than the initial depletion. Only the cod stock failed to recover, still showing SSB values below MSY B_trigger_. Eventually, we also observed non-stationarity in the important stock-recruitment relationship (SRR) in all species. In total, our study delivers further support for non-linear population dynamics to prevail in commercially exploited fish species^2,10^. A consequence of this results is that fisheries management cannot rely on common linear dynamic assumptions, but rather has to deal with delayed or failed recovery after implementing measures to reduced exploitation pressure as well as surprising developments such positive tipping.

We found positive tipping dynamics in the North sea stocks of plaice and hake. These stocks recovered the fastest in response to reduced fishing mortality after the reform of the CFP and increased to record high stock sizes recently. Such dynamics can be interpreted as positive tipping, where the pressure is reduced to a point (here below F_MSY_) that positive runaway dynamics in the population is induced. Positive tipping is in contrast to the general connotation of tipping points that are mainly seen as negative transitions in dynamic systems implying a shift from a positive to a negative state^2,10^. However, it should be noted that the increases in both stocks are spatially-explicit phenomena. The increase in North Sea plaice as a whole is due to a strong increase in stock components north of Scotland as well as the offshore areas of the Netherlands, Germany and Denmark^31^. The increase of hake in the North Sea is the result of stronger influx of individuals from the western distribution area of the northern stock component^32^. Nevertheless, both stocks increased due to reduced exploitation pressure and are important examples of the effectiveness of the MSY approach of the CFP. Such unprecedented stock sizes may however induce new management challenges such as shown for hake^32^.

In strong cases, regime shift dynamics with strong hysteresis and non-stationary SRR can lead to a failure to achieve management measures with cod as the best example^2,10^. The North Sea cod stock appears to be locked in a low SSB state as indicated by an open hysteresis loop. Our results suggest that cod crossed a tipping point (in this case a negative one) after which recovery is difficult. Cod recovery is hindered by the detrimental effects of climate change and especially warming^2,9^. However, it remains clear that cod recovery in the North Sea is also limited by fishing mortalities still above the F_MSY_ target.

In addition, our tGAM analyses revealed a significant thermal threshold in the SRR of saithe. The mean SST for the North Sea over the past 20 years was at 10.8°C^33^, exceeding the tGAM threshold identified (10.2 °C). The region records a temperature increase especially since the 1980s and mainly in the south-east^34^. Even though our analyses suggested no tGAM and therefore no temperature threshold for cod, increasing temperature effects are known for causing discontinuity in North Sea cod stock dynamics^2,9^. As temperature is expected to increase further due to climate change, it is assumed that effects on temperature vulnerable species’ SRR will remain or become even more severe^35^. No significant thermal thresholds were found for plaice and hake, indicating that these species likely can better cope with increasing temperatures as also reflected in their positive tipping dynamics.

We based our analyses on fisheries stock assessment data that have known limitations, while it is widely recognized that analysing these data provides new relevant understandings about fish stock dynamics^2,9,10^. With this database at hand we explored recovery patterns of fish stocks in the North Sea after the recent reform of EU’s CFP, implementing MSY principles^1^. Our study revealed all three regime shift characteristics to exist in all six North Sea fish stocks investigated, suggesting the prevalence of discontinuous dynamics in exploited living marine resources. Our results furthermore demonstrate the dependence of such dynamics on the environmental context and hence the ability of management measures to rebuild depleted fish stocks.

## Methods

### Data

We based our analyses on stock assessment data for six North Sea fish species (plaice, hake, herring, haddock, saithe and cod) provided by the International Council for the Exploration of the Sea (ICES) in the ICES Stock Assessment Database (data extracted in 2021, see Supplementary Table S4)^18^. Data for plaice comprised the years 1957–2021, for hake 1978–2021, for herring 1947–2021, for haddock 1972–2021, for saithe 1967–2021, and for cod 1963–2021. ICES stock assessment data include, among others, yearly data on spawning stock biomass (SSB), recruitment (R), and fishing mortality (F). R is represented by the population numbers at a certain age, i.e. for plaice at age 1, hake at age 0, herring at age 0, haddock at age 0, saithe at age 3, and cod at age 1.

Yearly mean sea surface temperature (SST) data for the North Sea region were derived for the tGAM analysis (see below) from the National Center for Environmental Information (NCEI)^33^.

### Approach

We here used three characteristics (flags) of regime shift dynamics in analysing the recovery of North Sea fish species: (1) abrupt shifts, (2) hysteresis, and (3) and non-stationary functional relationships^11–13^. We first identified abrupt changes in time-series of SSB using statistical change point analysis. Since regime shifts theoretically imply alternative stable states and these can be empirically assessed by exploring hysteretic dynamics, we assessed hysteresis in fish stocks by in inspecting the temporal evolution of the relationship of F to SSB. If hysteresis exists usually important functional relationships in a system are non-stationary and multiple drivers interact. We therefore analysed the most important functional relationship in a fish stock, the so-called stock-recruitment relationship (SRR) using multiple statistical techniques.

### Statistical change point analysis for detecting abrupt shifts

We identified abrupt changes in time series of SSB for each fish stock using statistical change point approaches^10^ provided by the R packages *bcp*^36^ and *changepoint*^37^. *bcp* calculates posterior probabilities of changes at any given point of the time series using a Bayesian approach^36^. We furthermore used the *BinSeg* algorithm in *changepoint*, that conducts binary segmentation based on a multiple change point search^37^. We determined years of abrupt changes when both methods detected approximately the same change point year (± 1 year). We allowed for at least five consecutive years between change points to potentially reflect quasi-stable periods in SSB.

### Hysteresis

We inspected hysteresis patterns in plots of F versus SSB for each North Sea stock analysed. As the effect of fishing pressure does not necessarily affect SSB instantaneously, we first used a cross-correlation to account for possible time lags (F_t−n_), using the ‘ccf’ function from the *stats* package^38^ in R. A first indication of hysteresis is visible by a loop-like shape appearing when the recovery path of SSB in response to reduced F differs from the initial path to a more depleted SSB state. This loop-like shape indicates that for the same level of the driver alternative stable states exist in the state variable. To statistically test these different paths for the indication of hysteresis, we applied breakpoint analyses using the R package *strucchange* (function ‘breakpoint’)^10,39^. In the *strucchange* approach, no a priori break points need to be determined. Deviations from linear regression models are tested for stability. Further, *m* breakpoints are assumed to exist and coefficients can shift between stable regression relationships, where *m* + 1 segments have constant coefficients. Minimizing the residual sum of squares (RSS) leads to the optimal number of breakpoints^39^.

### Non-stationary functional relationships

We investigated non-stationarity in the so-called stock-recruitment relationship (SRR) that relates the number of new offspring in a year (i.e. the recruitment; shifted to the year of origin) to the size of the parent spawning stock biomass (SSB). For each of the six North Sea fish stocks analysed we conducted a model selection exercise comparing traditional continuous Beverton–Holt and Ricker functions to alternative breakpoint approaches as well as a linear model. Beverton–Holt and Ricker models were fitted using the R package *FSA*^40^.

The Beverton–Holt and Ricker functions are given as followed:

Beverton–Holt^41^:

1 $$R = \frac{{\left( {\alpha *SSB} \right)}}{{\left( {1 + \beta *SSB} \right)}}$$

Ricker^42^:

2 $$R = SSB*e^{\alpha - \beta *SSB}$$

where R is the recruitment, SSB the spawning stock biomass, α and β parameters of the models^40^.

Discontinuous models incorporating breakpoints between different linear "sub-models" were fitted with algorithms provided by the R packages *segmented*^43^ (function ‘segmented’) and *strucchange*^39^ (function ‘breakpoint’). In *segmented*, ‘segmented’ linear models (LM) or generalized linear models (GLMs) are fitted to portions of the data points separated by breakpoints that need to be determined a priori. New linear relationships are estimated at each break point based on breakpoint and slope parameters. If these regressions are significantly different, a break point is found^43^. We compared the fits of a simple linear model, a linear model with logarithmic transformation of both variables (SSB and R) and a number of GLMs assuming Gaussian, Poisson, quasi-Poisson and negative binomial residual distributions. The best performing GLM was chosen based on model diagnostics and on over- or underdispersion patterns in the residuals. We here assumed only one breakpoint and used the mean of SSB as a starting value. We compared the best breakpoint models with the Beverton–Holt and Ricker as well as a simple linear model performing a leave-one-out cross-validation and calculating the root mean square errors (RMSE) for the test and the training data set^44^, where lowest RMSE for the training data set indicates the best fitting stock-recruitment model. As we were looking for break points in the past and therefore existing data, we focused on the training data set, since the test data set evaluated the performance for predictions.

### Temperature influence on stock-recruitment relationships

To assess whether abrupt changes in the relationship between SSB and R are affected by changes in sea surface temperature (SST), we used the threshold generalized additive modelling (tGAM) approach provided by the R package ‘INDperform’^21^. tGAMs are based on the following generalized additive model (gam):

3 $$gam\left( {SSB \sim s\left( {R,k = 3} \right)} \right)$$

where k is the dimension of the basis functions for representing the smooth term defined by s().

The SST threshold is identified by fitting the GAM with the ‘thresh_gam’ function, which uses the following formula^21^:

4 $$y \sim 1 + s\left( {SSB,by = {\rm I}\left( {1*\left( {R \le threshold} \right)} \right),k = 4} \right) + s\left( {SSB,by = {\rm I}\left( {1*\left( {R > threshold} \right)} \right),k = 4} \right)$$

The model estimates a threshold value for temperature from the input data by minimizing generalized cross-validation (GCV) values over an interval between lower and upper quantiles of the threshold variable. The quantile values are per default 0.2 for the lower and 0.8 for the upper quantile^21^. Using the ‘thresh_gam’ function, a sequence of evenly distributed threshold values between the defined lower and upper quantile is created. Along the sequence, a threshold GAM is implemented for every value at which a new splitting of threshold variables occurs.

For all observations where the threshold variable is below the threshold value at a certain time step (year), a smoothing function is applied. Another smoothing function is applied to the observations where the variable is higher than the threshold value. From several computed models, the tGAM with the least GCV is selected and its threshold value returned^21^. In the next step, we applied the ‘loocv_thresh_gam’ function (INDperform^21^) for the Leave-One-Out Cross-Validation (LOOCV) procedure to compare the fit of the returned tGAM with its corresponding GAM. In this way, the choice for or against a tGAM model was made. If the LOOCV revealed a tGAM, the choice for or against a true temperature threshold was based on three model outputs: (1) the effective degrees of freedom (edf) before and after the threshold had to differ (edf are a proxy for the degree of non-linearity; edf = 1 represent a linear relationship, edf > 1 and ≤ 2 show a weakly non-linear relationship, edf > 2 indicate a strongly non-linear relationship^45^), (2) at least one of the slopes needed to be significant (significance level *p* < 0.05), (3) the GCVV plot displaying GCV values of fitted tGAMs (y-axis) had to show a deep valley at the proposed threshold temperature (x-axis). Temporal autocorrelation was assessed using ACF plots. To see a possible difference of the SRR before and after the threshold, the SRR was predicted using the respective tGAM, predicted data were plotted and divided into red (high temperature) and blue (low temperature)^21^.

### R environment and packages

All analyses were conducted within the R programming and statistical environment^38^. For graphics the packages ‘ggplot2’^46^ and ‘patchwork’^47^ were used. An overview of packages and functions used in the analyses described above can be found in Table 2.

**Table 2:** R packages and functions used in the North Sea stocks analyses.

Analysis	Package	Function
Change/break points	bcp^35^	bcp()
	changepoint^36^	cpt.mean()
	segmented^40^	segmented()
	strucchange^41,45^	breakpoints(), breakfactor()
Lag F-SSB relationship	stats^38^	ccf()
Beverton–Holt	FSA^37^	srStarts(), srFuns()
Ricker	FSA^37^	srStarts(), srFuns()
GLM	MASS^46^	glm.nb()
tGAM	INDperform^20^	thresh_gam(), loocv_thresh_gam()

## Supplementary Information

Supplementary Information.

## Data Availability

The stock assessment data we used are from the International Council for the Exploration of the Sea (ICES), and are freely downloadable from the ICES Stock Assessment Database, available at https://standardgraphs.ices.dk/stockList.aspx. The temperature data are freely available from NOAA/ESRL PSD at https://psl.noaa.gov/data/gridded/data.noaa.ersst.v4.html. All data and codes are freely available from github.com/HeleneGutte/tipping_northsea_fish.
