# Supplementary material for: Regime shift dynamics, tipping points and the success of fisheries management
Source: Sci Rep. 2023 Jan 6;13:289. doi: 10.1038/s41598-022-27104-y (PMC9822959; doi:10.1038/s41598-022-27104-y)
Supplement: Supplementary file 1 — Supplementary Information. [file 41598_2022_27104_MOESM1_ESM.pdf]

Supplementary Information

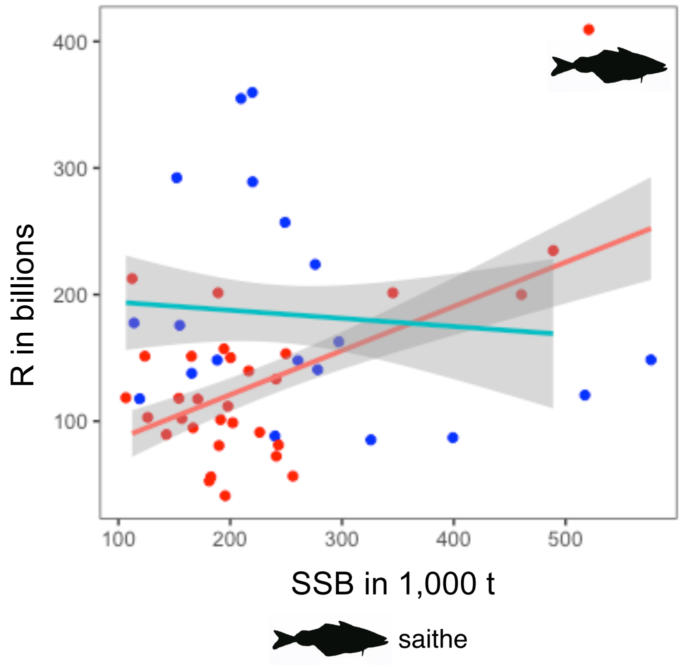

Supplementary Figure S1. Threshold GAM model for saithe. Model outcomes showing stock-recruitment relationship values before (blue) and after (red) the threshold. Solid line represents predicted tGAM model data using a lm-smoother for trend visualization. In grey the confidence interval of the line is shown. Points show original data. Spawning stock biomass (SSB), recruitment (R).

Supplementary Table S1. All change and break points detected for all species. SSB – spawning stock biomass, R – recruitment, F – fishing mortality

Species	Variable/ Function	Change/break point 1	Change/break point 2	Change/break point 3	Change/break point 4
Plaice	SSB	1969	1991	2007	
	SSB vs F	1992	2008		
	R vs SSB	1999			
Hake	SSB	1985	2010		
	SSB vs F	1993	2008	2014	
	R vs SSB	2008			
Herring	SSB	1966	1983	2000	
	SSB vs F	1957	1968	1983	2000
	R vs SSB	1983			
Haddock	SSB	2001			
	SSB vs F	1989	2003		
	R vs SSB	2013			
Saithe	SSB	1975	2010		
	SSB vs F	1973	1983	1996	
	R vs SSB	2006	2011		
Cod	SSB	1972	1999		
	SSB vs F	1972	1983	1999	2007
	R vs SSB	1996			

Supplementary Table S2. Root Mean Square Error (RMSE) of stock-recruitment model fits. Bold highlighted RMSE values represent best fitting models with lowest RMSE.

Species	Data set	Linear	Beverton-Holt	Ricker	Segmented	Seg-mented loga-rithmic	Segmented negative binomial	Strucchange
Plaice	Test data	428348	413424.9	**412932.6**	444398.5	1136697	1136697	*
	Training data	622899.2	634924	635932	**615763.5**	1299206	1299205	*
Hake	Test data	105030.5	**	111169.6	102912.6	351309.2	351309.2	**37305.04**
	Training data	132498.8	**	137915.1	122850.2	375887.9	375887.9	**26700.09**
Herring	Test data	11840339	**10382302**	11360380	10523814	30090531	30090531	12401912
	Training data	14644301	14054794	14145483	**12489186**	33695711	33695711	15120261
Haddock	Test data	353472.3	**327969.1**	328159.6	367786.2	635500.7	635500.5	605544.2
	Training data	484708.8	502865.2	502832.9	437547.6	880386	880385.8	**433818.3**
Saithe	Test data	**61203.27**	**	61337.45	64596.69	148081.2	148081	66347.47
	Training data	78700.1	**	82798.65	77814.61	168906.1	168906	**75644.11**
Cod	Test data	380892.3	**355558.5**	356373.9	387533.6	681643.2	681643	607106.1
	Training data	484346.4	502615.5	502577.7	437195.9	879697.3	879697.1	**433741.6**

*no significant breakpoint model.

**no Beverton-Holt model could be evaluated as no starting values could be fitted.,

Supplementary Table S3. Results threshold generalized additive modelling. The model diagnostics include the comparison of the effective degrees of freedom (edf) and the p values, which each need to be different from each other to accept the threshold. *p*-values < 0.05 were considered statistically significant.

Species	n	edf	*p*-value	Temperature threshold (°C)	Threshold accepted
Plaice	No tGAM (Leave-One-Out Cross-Validation (LOOCV))				
Hake	No tGAM (Leave-One-Out Cross-Validation (LOOCV))				
Herring	No tGAM (Leave-One-Out Cross-Validation (LOOCV))				
Haddock	No tGAM (Leave-One-Out Cross-Validation (LOOCV))				
Saithe	50	3.531	0.000	10.2	Yes
		2.67	0.000		
Cod	No tGAM (Leave-One-Out Cross-Validation (LOOCV))				

Supplementary Table S4. Overview of data used in analyses. Ranges of fishing mortality (F), spawning stock biomass (SSB), recruitment (R), sea surface temperature (SST).

Species	F	SSB (1,000t)	R (billions)	SST (°C)	Assessment period
Plaice	0.42-0.71 (ages 2-6)	213.890 – 1019.257	0.358303-4.434690 (age 1)	9.2-11.58	1957-2021
Hake	0.24-1.17 (length 50-80cm)	23.231-307.092	1.40670-7.72351 (age 0)	9.2-11.58	1978-2021
Herring	0.07-1.42 (ages 2-6)	105.793-5304.809	2.523740-69.493500 (age 0)	9.2-11.58	1947-2021
Haddock	0.18-0.96 (ages 2-4)	51.828-550.753	0.065563-50.765467 (age 0)	9.2-11.58	1972-2021
Saithe	0.3-0.73 (ages 4-7)	106.632-576.387	0.041095-4.09407 (age 3)	9.2-11.85	1967-2021
Cod	0.37-1.16 (ages 2-6)	31.978-219.8379	0.067402-2.370072 (age 1)	9.2-11.85	1963-2021
